# Effect of Endotoxemia Induced by Intraperitoneal Injection of Lipopolysaccharide on the Mg isotopic Composition of Biofluids and Tissues in Mice

**DOI:** 10.3389/fmed.2021.664666

**Published:** 2021-07-23

**Authors:** Rosa Grigoryan, Marta Costas-Rodríguez, Elien Van Wonterghem, Roosmarijn E. Vandenbroucke, Frank Vanhaecke

**Affiliations:** ^1^Atomic & Mass Spectrometry – A&MS Research Unit, Department of Chemistry, Ghent University, Ghent, Belgium; ^2^Vlaams Instituut voor Biotechnologie (VIB) Center for Inflammation Research, Ghent, Belgium; ^3^Department of Biomedical Molecular Biology, Ghent University, Ghent, Belgium

**Keywords:** endotoxemia, LPS-injected mice, Mg isotope fractionation, isotope ratio, minor elements, multi-collector ICP-mass spectrometry

## Abstract

Endotoxemia induced *in vivo* in mice by intraperitoneal injection of lipopolysaccharide (LPS) leads to (neuro)inflammation and sepsis. Also the homeostasis of mineral elements can be altered through mechanisms that still are poorly understood. The isotopic composition of Mg and the concentrations of the minor elements Ca, K, Mg, Na, P, and S were determined in biological fluids and tissues of young (14–28 weeks) and aged (40–65 weeks) LPS-injected mice and age-matched controls to reveal potential effects of the LPS-induced infection. Blood plasma of young and aged LPS-injected mice showed a heavy Mg isotopic composition, as well as elevated Mg and P concentrations, compared to matched controls. The plasma Mg isotopic composition was correlated with the P concentration in aged mice. Also the liver Mg isotopic composition was strongly affected in the young and aged LPS-injected mice, while for aged mice, an additional effect on the urine Mg isotopic composition was established. These observations were hypothetically associated with liver inflammation and/or hepatotoxicity, and reduced urinary Mg excretion, respectively. Also a regional endotoxin-induced difference was observed in the brain Mg isotopic composition for the aged mice only, and was attributed to potential disruption of the blood-brain barrier.

## Introduction

Lipopolysaccharide (LPS) is a structural component of the outer membrane (OM) of Gram-negative bacteria (GNB), which consists of three main components—lipid A (which is held responsible for the toxicity of the endotoxin), the core oligosaccharide, and the O-polysaccharide or O-antigen (1). Food, water and soil pathogens threatening human health include numerous Gram-negative bacteria, e.g., *Escherichia coli, Helicobacter, Legionella, Salmonella*, and *Yersinia* (2). The administration of LPS *in vivo* is a well-established experimental model of endotoxemia, inducing (neuro)inflammation and sepsis in mice (3). The LPS activates macrophages/monocytes, with the subsequent production and release of proinflammatory cytokines (e.g., IL-1, IL-6, and TNF-α) (4), thus inducing inflammation, mitochondrial damage and adenosine triphosphate (ATP) depletion (5). Various molecular changes have been found to occur under such circumstances, depending on the animal model, age and/or LPS treatment applied, i.e., a single vs. systemic dose, type of injection (subcutaneous, intraperitoneal, or intracerebroventricular), the LPS concentration administered and the duration of the treatment (6–8). In general, animals injected with LPS show increased levels of the LPS-binding protein (LBP) and develop conjunctivitis, diarrhea, lethargy, piloerection, and huddling symptoms (9, 10). The endotoxemia-induced toxicity also leads to injuries in several organs, particularly liver, kidneys, lungs, and brain (11). The liver plays a major role in the infection, as it takes up the infused LPS after the injection, rendering it the major localization of the LPS (12), and has the ability to clear the LPS to prevent it from entering into the systemic blood stream (13). In humans, LPS-induced hepatic injury has been reported in cirrhosis, autoimmune hepatitis, primary biliary cirrhosis and hepatitis C. As a result, detection and monitoring of endotoxemia can play an important role in evaluating the progression of such disease (14).

It is known that bacterial cells are capable of binding metals (15, 16), but the metal-binding properties of LPS in the body still need to be elucidated. It has been suggested that the sites responsible for the metal-binding of LPS are the negatively charged phosphoryl groups in lipid A (16). The OM is highly anionic, thus it might be also responsible for metal-binding in gram-negative bacteria (17). Magnesium is an important ingredient of the OM, which is responsible for neutralization of negatively charged phosphate residues in the lipid A and core regions (18). Hoyle and Beveridge (19) and Simpson et al. (20) suggested that the binding behavior of Mg^2+^ toward purified OM extracted from *Escherichia coli* might rely on its participation in cytoplasmic functions, involved in the synthesis of lipid A. Mg shows a hepatoprotective effect, which might be related to its anti-inflammatory, antioxidant and antiapoptotic properties (21). Magnesium sulfate treatment mitigated acute lung injury, inflammatory response, and oxidative stress in LPS-injected rats (22). Additionally, it has also been shown that the LPS administration activates several Transient Receptor Potential (TRP) cation channels (for Ca^2+^, K^+^, Na^+^, and Mg^2+^), inducing electrochemical gradients across the cell membrane with subsequent molecular and structural rearrangements (23–25). Disruptions of Mg^2+^ metabolism are associated to infections and immune dysfunction (26). Also, the change in Mg status observed upon aging may be due to insufficient intake or alterations in the Mg metabolism. Mg deficiency is thought to contribute to aging and subsequent vulnerability of the immune system and age-related diseases (27, 28). As Mg is mainly an intracellular ion, determination of the total serum/blood Mg concentration does not adequately reflect the Mg status and therefore, various other markers have been suggested for this purpose (29).

Hypermagnesemia is the Mg metabolism disturbance commonly caused by decreased kidney function (30) and increased intestinal absorption. Ferrè et al. (31) suggested that urinary Mg^2+^ excretion may be insufficient to balance intestinal Mg^2+^ absorption, which may cause high plasma Mg^2+^ levels. LPS-induced hypermagnesemia was associated with decreased renal function rather than with enhanced intestinal absorption due to downregulated expression of genes and Mg^2+^ transporting proteins involved in paracellular and transcellular cation transport across the intestine (3).

Biological processes are known to have the ability to fractionate the isotopes of essential metals. As a result, changes in the isotopic composition of these elements can be used to detect disruptions in metal metabolism. Although the Mg isotopic composition has so far been less explored than that of other metals (i.e., Ca, Cu, Fe, and Zn) for this purpose, such changes in the isotopic composition of Mg in human serum was observed in the pathology of diabetes type 1 (32). Characterization of the body distribution of the Mg isotopes may also be valuable for a better understanding of the disrupted Mg metabolism in LPS-induced endotoxemia. *In vitro* studies using *E. coli* bacteria examined the Mg isotope fractionation between cells and their growth medium. The cells showed a heavy Mg isotopic composition (enriched in the heaver Mg isotopes) with respect to the culture medium (33). Recently, it has been demonstrated that some bacteria have a preference for the heavier Mg isotopes for producing ATP in their cells (34).

In this work, potential alterations of the Mg isotopic composition and of the concentrations of the minor elements Ca, K, Mg, Na, P, and S were investigated in a murine model of endotoxemia to examine the effect of the LPS-induced infection in the body. Young (14–28 weeks) and aged (40–65 weeks) mice were injected intraperitoneally with a single dose of LPS and sacrificed 24 h after administration. As controls, age- and gender-matched mice injected with a saline solution were used. Mg isotopic analysis of mice tissues, including different brain regions, and fluids (plasma and urine) was accomplished using multi-collector ICP-mass spectrometry (MC-ICP-MS), while for the determination of the minor elements single-collector sector-field ICP-MS was used.

## Experimental

### Animals

Male young (14–28 weeks) and aged (40–65 weeks) wild type (C57Bl/6, APP^NLGF^, Ccl2-RFP^fl/fl^ backgrounds) mice were used in this experiment. The animals were housed at the VIB (Flemish Institute for Biotechnology)—UGent Center for Inflammation Research (IRC) in air-conditioned, stable climatic facilities (room temperature 21 ± 1°C, relative air humidity 60%), with natural day/night cycles of 14 h light and 10 h of darkness, with food and water *ad libitum*. All experiments were approved by the Ethical Committee of Animal Welfare of the Faculty of Sciences at Ghent University.

Mice were randomly assigned before starting the experiments. One group of young animals and another group of aged animals received *Escherichia coli* LPS in a single dose of 10 mg/kg b.w. in a phosphate-buffered saline solution (PBS) *via* an intraperitoneal injection. Age- and gender-matched controls were treated correspondingly with the pure PBS solution. The volume of the LPS and the PBS solutions injected were similar and adjusted for the body weight of each individual animal. Twenty-four hours after LPS administration, all animals were sacrificed under anesthesia. Body temperature and weight were monitored before and 24 h after the LPS-injection. The LPS administration causes a decrease in body weight and body temperature for young and aged mice. Individuals without decreased body weight and temperature after the LPS-injection were excluded from the experiment as this reflects a failed/suboptimal intraperitoneal injection.

Urine was sampled directly from the bladder of sedated mice. After sacrification and perfusion of the animals, other biofluids and tissues were collected and stored at −20°C until sample preparation in a class-10 clean lab (PicoTrace™, Göttingen, Germany).

### Sample Preparation

A total of 172 samples were digested using trace metal analysis grade PrimarPlus 14 M HNO_3_ (further purified using sub-boiling distillation) and ultra-pure TraceSELECT^®^ 9.8 M H_2_O_2_. A mixture of 2 mL of HNO_3_ and 0.5 mL of H_2_O_2_ was used for digestion of the fluids (blood plasma and urine) and a mixture of 4 mL of HNO_3_ and 1 mL of H_2_O_2_ was used for the tissues, bone and food. The sample digestion was accomplished in closed Savillex^®^ PFA beakers, heated at 110°C for 18 h on a hotplate. The sample digests thus obtained were evaporated until dryness at 90°C and re-dissolved in 1.2 mL of 0.4 M HCl. An aliquot of each sample digest was used for the quantitative determination of the aforementioned minor elements and the remaining sample digest was used for Mg isotopic analysis. Pure Mg fractions are required for accurate and precise isotope ratio measurements using MC-ICP-MS, as only then the bias caused by instrumental mass discrimination can be adequately corrected for. Thus, Mg was chromatographically isolated from the sample matrix using 1 mL of AG50W-X8 cation exchange resin (100–200 mesh, hydrogen form), as described in ref. (32). Mg fractions from bone samples showing a Ca/Mg ratio ≥ 1.5 after purification, were subjected to a second chromatographic purification following the same procedure (32).

### Determination of the Mg Isotope Ratios

Mg isotope ratios were measured using a Neptune XT MC-ICP-MS instrument (Thermo Scientific, Bremen, Germany) equipped with a high-transmission Jet interface (Jet-type Ni sampling cone and X-type Ni skimmer cone). The solutions were introduced into the ICP using a 100 μL min^−1^ PFA concentric nebulizer mounted onto a double spray chamber with cyclonic and Scott-type sub-units. Measurements were performed at pseudo-medium mass resolution (R = 4,000), in static collection mode and using three Faraday collectors connected to 10^11^ Ω amplifiers, as described in refs. (32, 35). Correction for instrumental mass discrimination was accomplished by relying on an external standard, measured in a sample-standard bracketing approach (SSB). Mg isotope ratios were measured at a concentration of 150 μg L^−1^ Mg in 0.28 M HNO_3_. The concentrations of samples and standard were matched within ± 5%. The Mg isotopic composition is expressed in delta notation (per mil, ‰) relative to the DSM3 international reference material. The δ^26^Mg value represents the relative deviation of the Mg isotopic composition of the sample (^26^Mg/^24^Mg)_sample_ vs. that of the DSM3 reference material (^26^Mg/^24^Mg)_DSM3_, calculated as indicated in Equation (1).

(1) $$\delta^{26}Mg_{DSM3}=\left(\frac{{\left(\frac{{\;}_{\;}^{26}Mg}{{\;}_{\;}^{24}Mg}\right)}_{sample}}{{\left(\frac{{\;}_{\;}^{26}Mg}{{\;}_{\;}^{24}Mg}\right)}_{DSM3}}-1\right)\;\;\times \;\;1000$$

### Quantitative Determination of Minor Elements

The quantitative determination of Ca, K, Mg, Na, P, and S was accomplished using a Thermo Scientific Element XR single-collector sector-field ICP-MS instrument. All solutions were introduced into the plasma using a 200 μL min^−1^ quartz nebulizer mounted onto a cyclonic spray chamber. For quantification, a 5-point calibration curve was relied on, while Ga was used as an internal standard, compensating for signal instability and matrix effects. A multi-element standard solution was prepared from single-element standard stock solutions from Merck (Darmstadt, Germany), PlasmaCAL (Quebec, Canada), Inorganic Ventures (Christiansburg, VA, USA), Alfa Aesar GmbH (Karlsruhe, Germany), and CPI International (Santa Rosa, CA, USA) of 1,000 mg L^−1^ (Ca, Mg, P, and S) or 10,000 mg L^−1^ (Na, K) and used for quantification purposes. The signals for the nuclides ^44^Ca, ^24^Mg, ^23^Na, ^31^P, and ^32^S were measured at medium mass resolution (R = 4,000) and that for ^39^K at high mass resolution (R = 10,000). Samples, standard solutions and procedural banks were appropriately diluted in 0.28 M HNO_3_.

### Statistical Analyses

IBM^®^ SPSS Statistics 26 software (SPSS Inc. Chicago, Illinois, USA) was used for statistical analysis of the data. Data were tested for normal distribution using the Shapiro-Wilk normality test. The non-parametric Kruskal–Wallis and Mann-Whitney *U*-tests and the parametric independent *t*-test were used for comparison of the results when appropriate. Bivariate analysis with a level of significance established at *p* ≤ 0.05 was used for assessing pairwise correlations.

## Results

The Mg isotopic composition and the elemental concentrations of Ca, K, Mg, Na, P, and S were determined in blood plasma, in body tissues, including different brain regions (brain stem, cerebellum, cerebrum, cortex, and hippocampus), and in urine of young and aged controls and LPS-injected mice. The groups of mice were denoted as follows: young control (Y, control), young LPS-injected mice (Y, LPS), aged control (O, control), and aged LPS-injected mice (O, LPS).

### Blood Plasma

**Figure 1:**
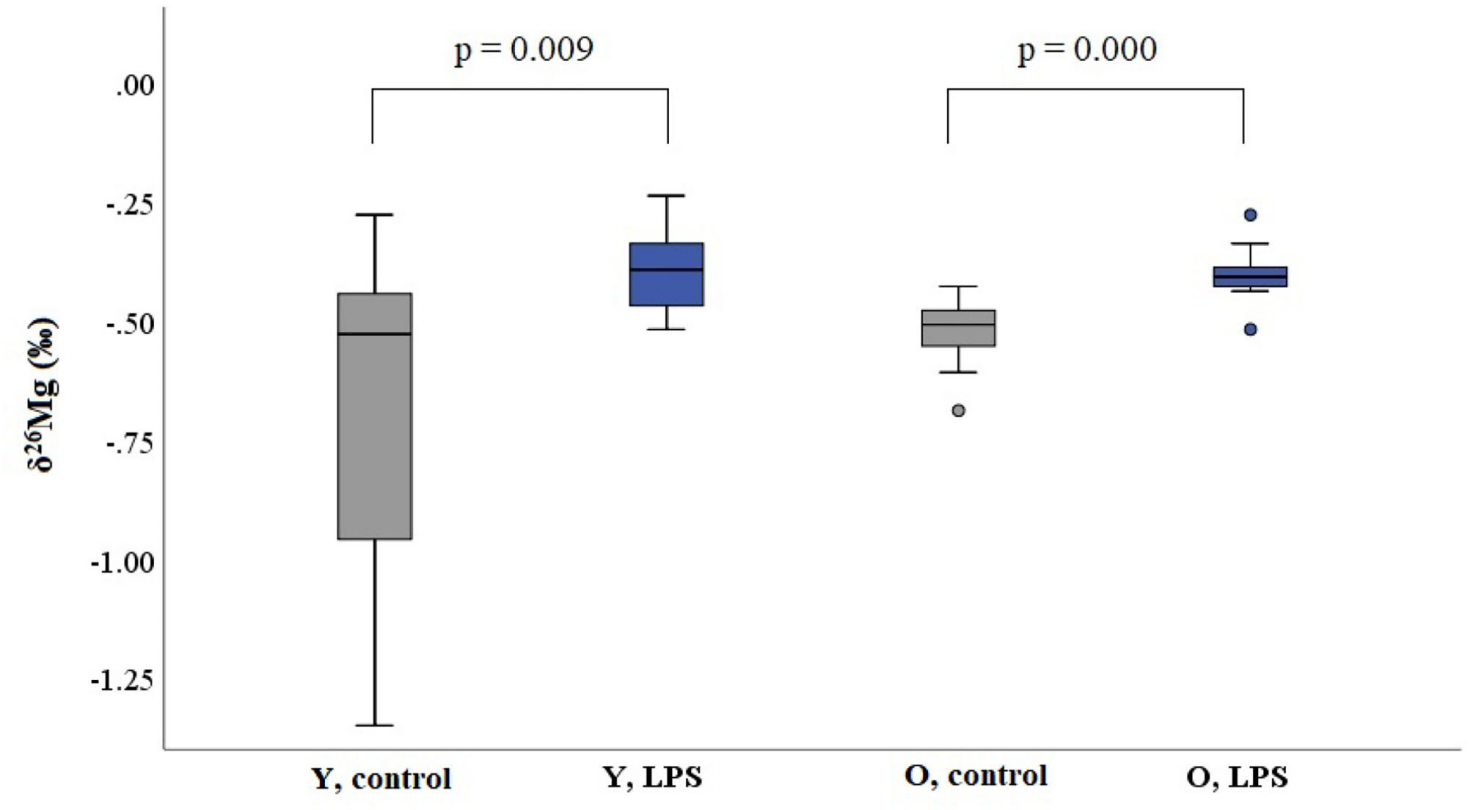
Boxplots for the blood plasma Mg isotopic composition for young and aged LPS-injected mice and the matched controls. Y and O correspond to young and aged mice, respectively. LPS corresponds to LPS-injected mice. These box plots compile the median, quartiles, and extreme values; individual dots are outliers.

Blood plasma samples from 47 individuals, i.e., 12 young controls, 10 young LPS-injected mice, 16 aged controls and 9 aged LPS-injected mice, were analyzed. Table 1 summarizes the Mg isotopic composition of the blood plasma for each group. The plasma Mg isotopic composition of the LPS-injected mice was significantly heavier (enriched in the heavier Mg isotopes) than that of the controls for both, young (Mann-Whitney *U*-test, *p* = 0.009) and aged (Mann-Whitney *U*-test, *p* = 0.000) mice. Figure 1 shows the box plots of the blood plasma δ^26^Mg values for the four groups. As can be seen, the isotopic variability within the group of the young controls was wide compared to that within the other groups. No significant difference could be established between the young and aged controls (Mann-Whitney *U*-test, *p* = 0.631).

**Table 1:** Mg isotopic composition of blood plasma for young (Y) and aged (O) LPS-injected mice and matched controls.

**Group**	**Control mice**					**LPS-injected mice**					***p*-value**
	**δ^26^Mg (‰)**					**δ^26^Mg (‰)**					
	**Mean**	**SD**	**Median**	**IQR**	***N***	**Mean**	**SD**	**Median**	**IQR**	***N***	
Y	−0.70	0.35	−0.53	0.53	12	−0.39	0.09	−0.40	0.15	10	0.009^**^
O	−0.53	0.07	−0.51	0.08	9	−0.40	0.07	−0.41	0.07	16	0.000^**^

*N is the number of individuals. IQR is the interquartile range*.

** *Significant difference between controls and LPS-injected mice at p < 0.01 (Mann-Whitney U-test)*.

Table 2 compiles the elemental concentrations of Ca, K, Mg, Na, P, and S in the blood plasma from the young and aged controls and LPS-injected mice. The Mg concentration in the plasma of the young LPS-injected mice was significantly higher (Mann-Whitney *U*-test, *p* = 0.014) than that in the matched controls, and the extent of this difference was even larger when comparing the aged LPS-injected group and the corresponding controls (Mann-Whitney *U*-test, *p* = 0.000). Also the concentration of P was significantly higher in the plasma of young and aged LPS-injected mice compared to that for the respective age-matched controls (for both the young and aged group: Mann-Whitney *U*-test, *p* = 0.000). The concentrations of Ca, K, Na, P, and S in blood plasma were similar for the young and aged LPS-injected mice and the respective age-matched controls (no significant differences). The difference in plasma concentration between young and aged controls was significantly relevant for S only (Mann-Whitney *U*-test, *p* = 0.000).

**Table 2:** Elemental concentrations (mg L^−1^) in blood plasma of young and aged LPS-injected mice and matched controls.

**Age**	**Element**	**Control mice**				**LPS-injected mice**				
		**Mean ± SD**	**Median**	**IQR**	***N***	**Mean ± SD**	**Median**	**IQR**	***N***	***P*-value**
Young mice	Ca	73 ± 14	75.5	27.5	12	66 ± 20	61.0	32.0	10	0.381
	K	970 ± 250	923	308	11	930 ± 280	902	421	10	0.654
	Mg	22.7 ± 4.3	21.5	4.75	12	28.1 ± 4.9	28.0	6.75	10	0.014^*^
	Na	2,730 ± 160	2,730	237	12	2,700 ± 270	2,765	456.3	10	0.872
	P	113 ± 16	116	22.0	11	200 ± 50	207	68.0	9	0.000^**^
	S^a^	263 ± 34	274	47.0	11	287 ± 25	282	24.3	8	0.091
Aged mice	Ca	71 ±11	69.5	14.0	14	62 ± 15	59.0	10.0	7	0.067
	K	900 ± 170	925	163	13	1,190 ± 500	1,062	937	8	0.547
	Mg	21.6 ± 2.2	22.0	3.25	14	33.0 ± 7.0	31.0	14.0	7	0.000^**^
	Na	2,920 ± 360	2,820	497	14	2,580 ± 380	2,700	596.0	7	0.287
	P	107 ± 25	104	41.5	13	200 ± 62	185	90.0	7	0.000^**^
	S^a^	322 ± 37	314	59.5	13	300 ± 120	321	155	8	0.860

*N is the number of individuals and IQR is the interquartile range*.

* *Significant difference between controls and LPS-injected mice at p < 0.05 (Mann-Whitney U-test)*.

** *Significant difference between controls and LPS-injected mice at p < 0.01 (Mann-Whitney U-test)*.

a *Significant difference between young and aged mice (p = 0.000, Mann-Whitney U-test)*.

Blood plasma Mg and P concentrations were correlated in both young and aged controls and LPS-injected mice (Spearman's rho coefficient, ρ = 0.675, *p* = 0.001 and ρ = 0.800, *p* = 0.000, respectively). However, only for the aged mice, a weak correlation was observed between the blood plasma Mg isotopic composition and the P concentration (Spearman's rho coefficient 0.459, *p* = 0.042) and no correlation was found between the plasma Mg mass concentration and its isotopic composition, suggesting that these hold different messages. A significant correlation was also found between the S and Na concentrations (Spearman's rho coefficient 0.583, *p* = 0.007) for aged mice.

### Body Tissues and Urine

The isotopic composition of Mg and the concentrations of Ca, Fe, Mg, Na, P, and S in body tissues and urine were investigated in the young and aged mice. A total of 94 samples, including food, bone (femur), intestine, kidney, liver, muscle, pancreas, and urine from young and aged controls (3 individuals for each age group) and LPS-injected mice (3 young individuals, 4 aged individuals) were analyzed. Results came from a fewer number of individuals than the numbers cited above due to limited sample availability for the elemental concentrations in urine for the aged controls (*N* = 2 instead of 3) and young LPS-injected mice (*N* = 2 instead of 3) and elemental concentrations in pancreas for aged controls (*N* = 2 instead of 3).

**Figure 2:**
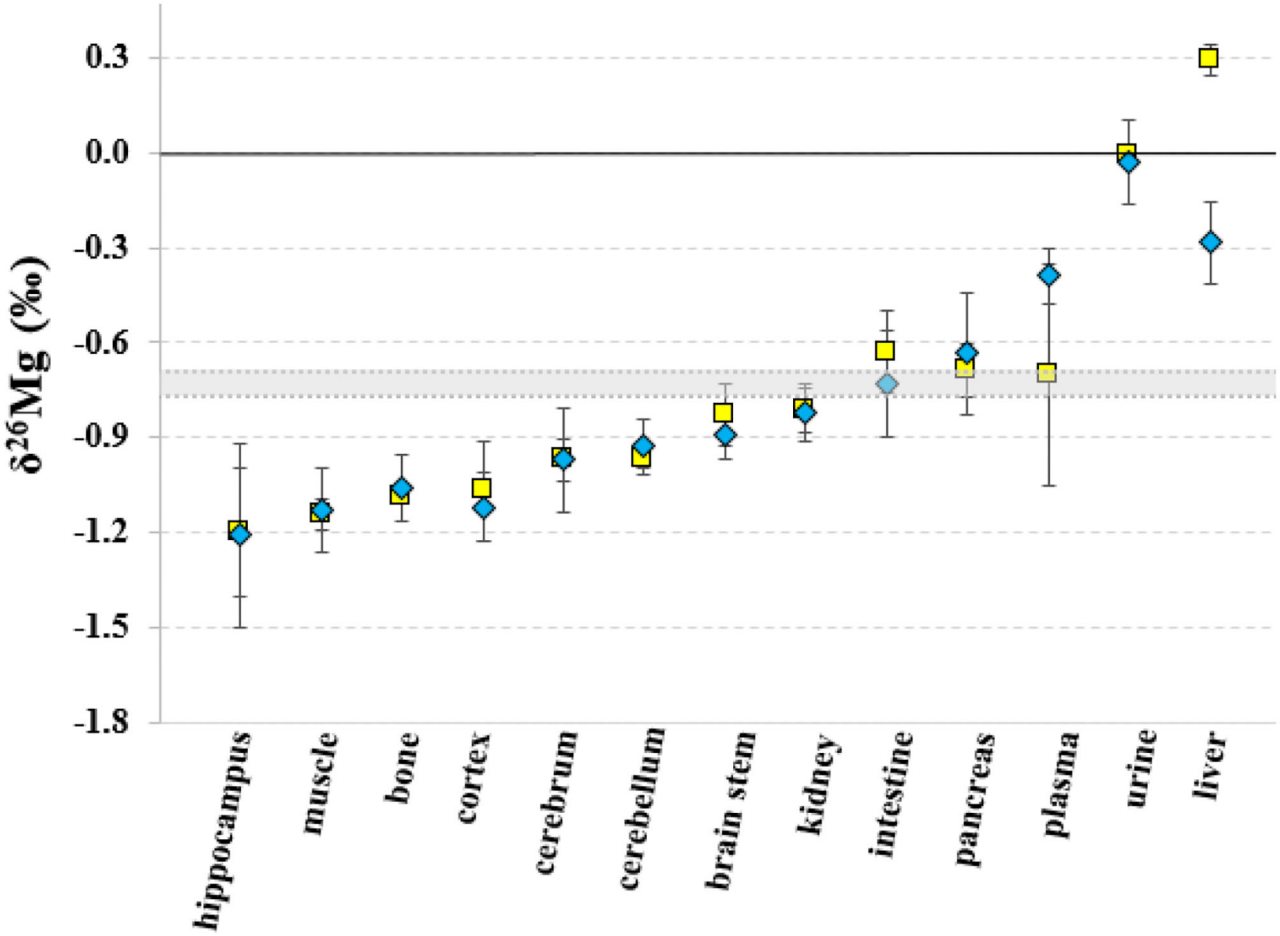
Body distribution of the Mg isotopes in young controls and LPS-injected mice. Yellow squares correspond to the control mice, blue diamonds to the LPS-injected mice and the gray area corresponds to the Mg isotopic composition of the food. Error bars indicate the standard deviation.

**Figure 3:**
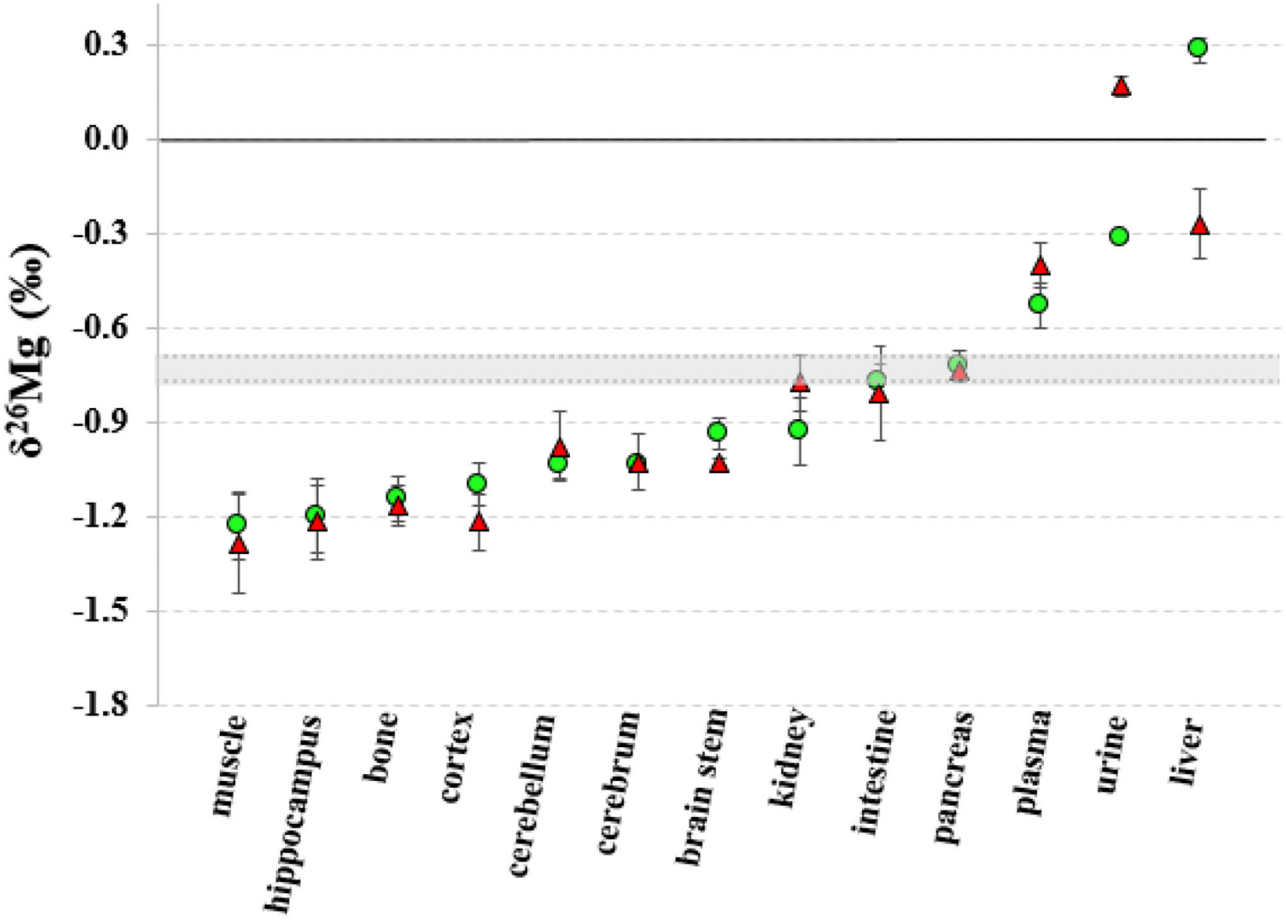
Body distribution of the Mg isotopes in aged controls and LPS-injected mice. Green circles correspond to the control mice, red triangles to the LPS-injected mice and the gray area corresponds to the Mg isotopic composition of the food. Error bars indicate the standard deviation.

The Mg isotopic composition of the food used for feeding all groups of mice ranged between −0.77 and −0.69 ‰ (*N* = 7), with an average δ^26^Mg value similar to the values for pancreas and intestine, which were not significantly different between controls and LPS-injected mice (Table 3). Figures 2, 3 show the body Mg isotopic distribution for the young and aged controls and LPS-injected mice. As can be seen, the liver Mg isotopic composition was significantly altered in the young LPS-injected mice. Also the aged LPS-injected mice showed a significant alteration in the liver and in urine Mg isotopic compositions.

**Table 3:** Mg isotope distribution in controls and LPS-injected mice.

**Sample**	**Controls**			**LPS-injected mice**			***p*-value**
	**δ^26^Mg (‰)**	**SD**	***N***	**δ^26^Mg (‰)**	**SD**	***N***	
Y, hippocampus	−1.20	0.20	3	−1.21	0.29	3	0.988
Y, cortex	−1.07	0.16	3	−1.12	0.11	3	0.696
Y, cerebrum	−0.97	0.03	3	−0.97	0.16	3	0.976
Y, cerebellum	−0.97	0.03	3	−0.93	0.09	3	0.505
Y, brain stem	−0.83	0.10	3	−0.89	0.08	3	0.465
O, hippocampus	−1.20	0.12	5	−1.22	0.12	5	0.870
O, cortex	−1.10	0.07	4	−1.22	-0.09	3	0.095
O, cerebellum	−1.04	0.04	7	−0.98	0.11	5	0.193
O, cerebrum	−1.04	0.02	3	−1.03	0.09	4	0.890
O, brain stem	−0.94	0.05	5	−1.03	0.01	3	0.015^*^
Y, muscle	−1.15	0.05	3	−1.13	0.13	3	0.847
Y, bone	−1.09	0.02	3	−1.06	0.11	3	0.691
Y, kidney	−0.81	0.07	3	−0.82	0.09	3	0.888
Y, intestine	−0.63	0.13	3	−0.73	0.17	3	0.473
Y, pancreas	−0.69	0.08	3	−0.64	0.19	3	0.642
Y, urine	−0.01	0.01	3	−0.03	0.13	2	0.721
Y, liver	0.29	0.05	3	−0.28	0.13	3	0.002^**^
O, muscle	−1.23^a^	0.10	3	−1.29	0.16	4	0.635
O, bone	−1.14^a^	0.07	3	−1.17	0.07	4	0.667
O, kidney	−0.93^a^	0.11	3	−0.78	0.09	4	0.087
O, intestine	−0.77^a^	0.06	3	−0.81	0.15	4	0.692
O, pancreas	−0.72^a^	0.05	3	−0.74	0.03	3	0.711
O, urine	−0.32^a^	0.01	2	0.17	0.03	3	0.002^**^
O, liver	0.28^a^	0.04	3	−0.27	0.11	4	0.000^**^
Food	−0.73^b^	0.04		−0.73^b^	0.04		

*The Mg isotopic composition is expressed as the δ^26^Mg value (mean and standard deviation). The number of individuals analyzed is N. Y is young and O is aged*.

a *Data from ref. (36)*.

b *All animals were fed with the same food. Mean ± SD (N = 4)*.

* *Significant difference between controls and LPS-injected mice at p < 0.05 (independent samples t-test)*.

** *Significant difference between controls and LPS-injected mice at p < 0.01 (independent samples t-test)*.

The liver Mg isotopic composition for the LPS-injected mice was on average 0.55‰ (*t*-test, p = 0.000) and 0.58‰ (*t*-test, *p* = 0.002) lighter than that of controls (*t*-test, *p* = 0.000) for aged and young mice, respectively, while the urine Mg isotopic composition of the LPS-injected aged mice was on average 0.49‰ heavier than that of age-matched controls (*t*-test, *p* = 0.002).

The concentrations of the minor elements fore aged and young mice are shown in the Supplementary Tables 1, 2, respectively. The Mg concentration in the intestine of the LPS-injected mice (167.0 ± 7.3 μg g^−1^) was significantly higher (*t*-test, *p* = 0.007) than that of controls (146.7 ± 3.8 μg g^−1^). Although the significance of the difference in the Ca, Mg, and Na concentrations of in the urine samples (aged LPS-injected vs. control mice) cannot be assessed due to the small sample size, it can be noted that the concentrations were reduced in the LPS-injected mice. The Mg concentration in the urine was significantly lower for the young LPS mice (*t*-test, *p* = 0.004) than for the corresponding controls. In other tissues, no statistical difference between controls and infected aged mice could be established. However, the Mg, P, and S showed a significant decrease of concentration in the kidneys of infected young mice, whereas the Ca concentration in liver of LPS-injected young mice was significantly increased (*t*-test, *p* = 0.007) compared to young controls.

### Brain Regions

The Mg isotopic composition was determined in the brain regions brain stem, cerebellum, cerebrum, cortex and hippocampus for the four groups of mice (young and aged LPS-injected mice and age-matched controls) to examine the potential effects of the LPS-induced infection and age on the brain. Each group consisted of 3–7 individuals, resulting in a total of 74 samples. Table 3 compiles the δ^26^Mg values for the brain regions.

**Figure 4:**
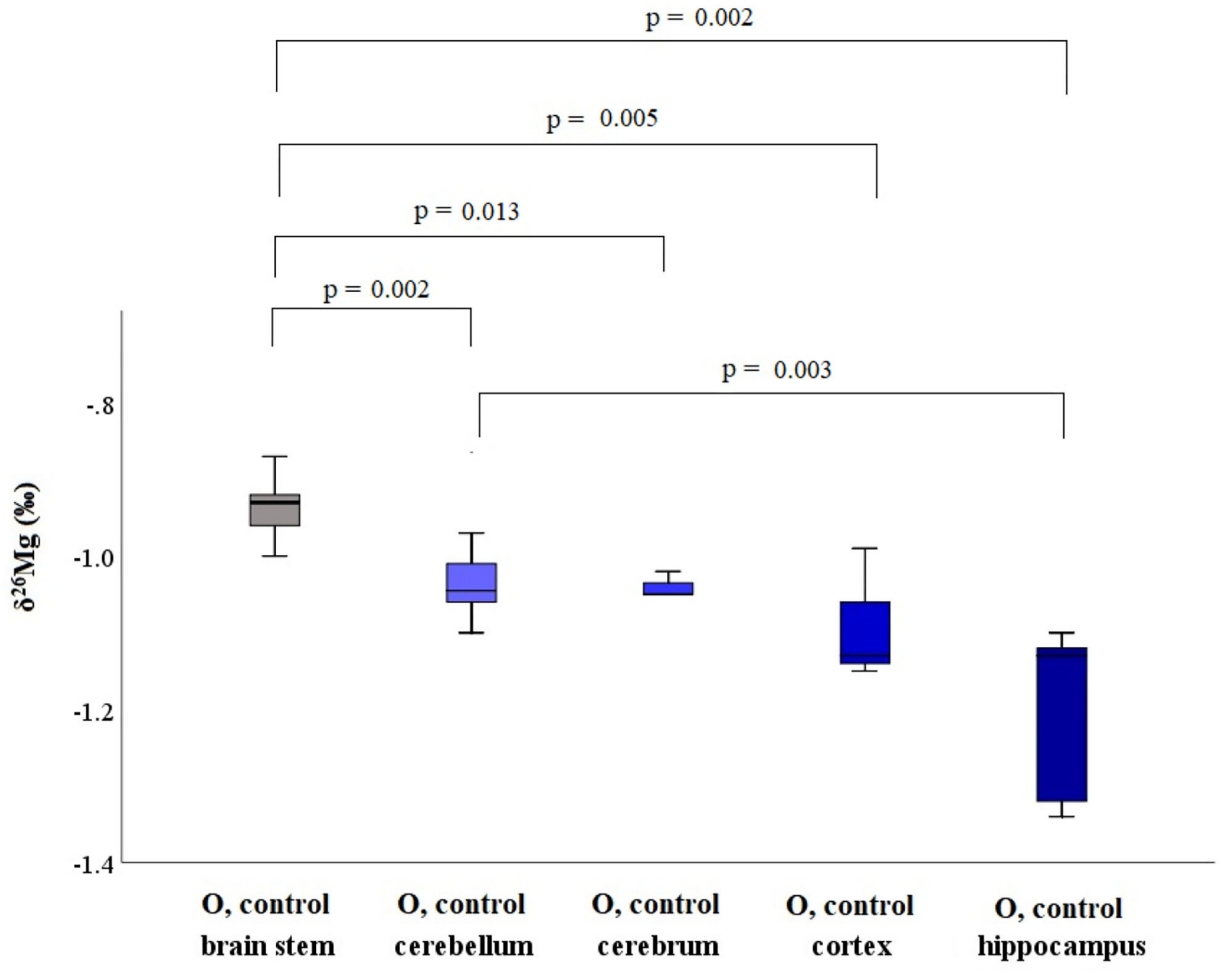
Boxplots for the Mg isotopic composition in different brain regions for aged controls. These box plots compile the median, quartiles and extreme values.

Within the brain of healthy mice, the δ^26^Mg values for different brain regions ranged between −1.20 and −0.83 ‰ (Table 3) and between the brain parts coming from a single healthy individual, the δ^26^Mg values ranged between −1.20 and −0.94 ‰ for aged mice and between −1.20 and −0.83 ‰ for young mice. The Mg isotopic distribution within the brain is illustrated for the aged controls in Figure 4. In the aged controls, the Mg isotopic composition varied significantly among regions (Kruskal-Wallis test, *p* = 0.002), but in the young controls no significant differences were found. The Mg isotopic composition of the brain stem was the heaviest and was significantly different from that of all the other regions in the aged controls. The hippocampus of the aged mice showed the lightest Mg isotopic composition, which was significantly different from that of the cerebellum (*t*-test, *p* = 0.003). When the brain regions of the young and aged control mice were compared, a significant age effect was observed for the cerebellum (*t*-test, *p* = 0.023).

LPS-injected mice showed a Mg isotopic distribution within the brain that is similar to that of controls. However, the brain stem Mg isotopic composition of the aged LPS-injected mice showed to be significantly lighter than that of the age-matched controls (*t*-test, *p* = 0.015).

Concentrations of Ca, K, Mg, Na, P, and S were also determined in the brain regions of the four groups of mice, resulting in a total of 68 samples. Data are summarized in the Supplementary Table 3. When young and aged controls were compared, it has been found that Mg and Na concentrations in the cortex from aged controls (136 ± 12 and 650 ± 110 μg g^−1^, respectively) were significantly lower than those in the cortex from young controls (155.3 ± 1.3 and 1,350 ± 170 μg g^−1^, respectively; *t*-test, *p* = 0.036 for Mg and *p* = 0.004 for Na), and that the Ca and K concentrations in the brain stem of aged controls (33 ± 13 and 1,930 ± 300 μg g^−1^, respectively) were significantly lower than those in the brain stem of the young controls (218 ± 55 and 2,620 ± 370 μg g^−1^, respectively) (*t*-test, *p* = 0.000 for Ca and *p* = 0.041 for K).

In general, the concentrations of these minor elements were not altered in the brain regions of the LPS-injected mice, with one exception: in the brain stem of the aged LPS-injected mice, the Ca, K and S concentrations (77.9 ± 7.0, 2,950 ± 430, and 920 ± 320 μg g^−1^, respectively) were significantly higher than those of the matched controls (33 ± 13, 1,930 ± 300, and 430 ± 120 μg g^−1^, respectively) (*t*-test, *p* = 0.007 for Ca, *p* = 0.014 for K, and *p* = 0.003 for S).

## Discussion

LPS is known to cause systemic inflammation, septic shock, renal failure and multi organ damage (4). The intraperitoneal administration of the LPS is a well-established model of endotoxemia inducing (neuro)inflammation (37). LPS administration induces the expression of pro-inflammatory markers in plasma and tissues of rodents (38).

Mg^2+^ is known to be crucial for the integrity of the bacterial OM and it also stabilizes LPS-LPS interactions. It binds to the anionic phosphate groups in the inner core, thus neutralizing them and stabilizing the OM (39, 40). We observed increased Mg and P concentrations in the blood plasma of both young and aged LPS-injected mice compared to matched controls, which might be caused by the disturbance of the Mg and P homeostasis induced by the LPS. In clinical practice, hypermagnesemia and hyperphosphatemia are accompanied by kidney disease, as a result of which urinary Mg and P excretion are reduced (30).

Body Mg homeostasis is mainly regulated by gastrointestinal absorption, bone (de)mineralization and renal excretion (41). The Mg concentration was also increased in the intestine and reduced in the urine of the aged LPS-injected mice compared to controls. The reduced urinary Mg^2+^ excretion may no longer balance the increased Mg^2+^ intestinal absorption, resulting in elevated plasma Mg^2+^ concentrations, i.e., hypermagnesemia. Meurer and Höcherl (3) suggested that the LPS-induced hypermagnesemia is mainly related to the reduced renal function in response to the LPS. The authors also found reduced intestinal Mg^2+^ absorption, caused by genes involved in paracellular cation transport across the small intestine, and the decrease of Mg^2+^ transporting proteins involved in the transcellular Mg^2+^ transport across the large intestine. However, also changes associated to intestinal LPS colonization, i.e., bacterial growth rates and locations in the intestinal mucus, can affect the Mg intestinal absorption (42, 43). Despite the low number of urine samples analyzed, the results pointed toward a decreased urinary excretion of Na in the LPS-injected mice as well, an observation that is in agreement with previous studies (3). However, further research is required with an increased number of urine samples.

In addition to the concentration of Mg in blood plasma, also its isotopic composition was significantly altered in the LPS-injected mice. However, no correlation was found between Mg concentration and isotopic composition, suggesting that they are revealing different biochemical information. Although little is known about the exact processes underlying Mg isotope fractionation occurring during biological processes, they are presumably governed by differences in metal binding and/or changes in the redistribution of Mg stores with distinct isotope compositions (16, 17).

Young and aged controls did not show a significant difference in plasma Mg isotopic composition, which is in agreement with our previous study, where no effect of age on the human serum Mg isotopic composition was observed (32). The blood plasma Mg isotopic composition of the LPS-injected mice was overall about +0.1‰ heavier than that of the matched controls, for both age groups (Table 1).

The isotopic composition of Mg in the liver, which shows the heaviest Mg isotopic composition of the body (Figure 2), was altered in the young and aged LPS-injected mice, while the Mg concentration did not show significant changes, compared to age-matched controls. The LPS-induced endotoxemia leads to a lighter hepatic Mg isotopic composition. The liver rapidly exchanges Mg with plasma (44) and responds promptly to the LPS with production of cytokines and reactive oxygen intermediates and clearance of bacteria (13). The urine Mg isotopic composition of the aged LPS-injected mice displays a reverse trend, showing an enrichment in the heavy Mg isotopes, which may result from the decreased urinary Mg excretion. The urine Mg isotopic composition of the young LPS-injected mice didn't show any significant difference compared to controls, as they are less sensitive to endotoxin exposure than aged mice (7). The intestinal Mg isotopic composition of the LPS-injected mice was similar to that of the matched controls, indicating that potential changes in the intestinal absorption in the LPS-injected mice did not induce any detectable effect on the corresponding Mg isotope fractionation. This is consistent with the LPS-induced hypermagnesemia associated to a reduced Mg excretion. Other organs or bone of the young and aged LPS-injected mice did not show a significant alteration of the Mg isotopic composition compared to matched controls. A single high dose of LPS does not induce changes in the Mg isotope fractionation affecting its isotopic composition in bone and organs, with the exception of the liver, 24 h after administration.

The isotopic composition of Mg showed to be heterogeneous within the brain (Figure 4), with the differences reaching statistical significance in aged controls. A shift toward a lighter Mg isotopic composition was observed in the cerebellum of the aged controls compared to young controls. In the young LPS-injected mice, none of the brain regions was affected by the endotoxin and the isotopic pattern was the same as that observed in controls. However, the brain stem of the aged LPS-injected mice showed a significantly lighter Mg isotopic composition than that of the controls, which can probably be related to the higher sensitivity of the aged mice toward exposure to LPS. Elevated cytokine levels, c-fos expression (indirect marker of neurological activity) and a reduced glutathione (GSH) concentration have been reported in the brain of LPS-injected mice (7, 45). In aged rats, systemic LPS injection induced prolonged neuroinflammation and astrocyte activation in the hippocampus (46) and in LPS-injected pigs, the pro-inflammatory cytokine IFN-γ level was elevated in the frontal cortex compared to saline-injected pigs (47). The lighter Mg isotopic composition found in the brain stem of the aged LPS-injected mice may result from the disruption of the blood-brain barrier (BBB) mediated by inflammatory and immune mechanisms (48). Mg concentrations, however, were not altered in the LPS-injected, either young or aged, mice, once more underlining the different messages in element concentrations and isotopic compositions, respectively. Na and S concentrations in the cortex and in the brain stem, respectively, of aged LPS-injected mice were elevated compared to matched controls. A decrease in Ca concentration was also observed for the brain stem of the young LPS-injected mice and in the cortex of the aged LPS-mice compared to the age-matched controls.

An age-related difference was observed for the blood plasma S concentration. Jeon et al. reported a significant decrease with age in plasma glutathione and hepatic homocysteine levels when comparing 2, 6, and 18 months old C57BL/6 male mice. While the level of the other sulfur-containing amino acids were not different among those age groups, the concentration of sulfur-containing amino acids was significantly affected in the liver of 30-months old mice (49). Interestingly, we observed a significant increase in the total sulfur blood plasma concentration in aged mice (40–65 weeks) compared to young controls (14–28 weeks), suggesting that total plasma sulfur concentration is not an indicator of the status of sulfur-containing amino acids in blood plasma.

## Conclusions

Blood plasma of young and aged LPS-injected mice showed a heavier Mg isotopic composition, together with elevated Mg and P concentrations, compared to matched controls. The Mg isotopic composition was not correlated with the Mg mass fraction, but showed correlation with the P concentration. ATP depletion induced by the LPS may contribute to an enrichment in the heavier Mg isotopes in the blood plasma. The hepatic response to the LPS-associated inflammation and/or hepatotoxicity and the reduced urinary Mg excretion seems to result in light hepatic and heavy urine Mg isotopic compositions in the aged LPS-injected mice, also leading to a heavy blood plasma Mg isotopic composition and hypermagnesemia. However, the Mg isotopic composition of the young mice was only altered in the liver as they are less sensitive to the endotoxin. Regional differences were observed in the brain Mg isotopic composition of healthy individuals. While the age did not significantly affect the plasma Mg isotopic composition, a shift toward a lighter Mg isotopic composition was observed in the cerebellum of the aged mice. The brain stem of the aged LPS-injected mice showed a lighter Mg isotopic composition compared to matched controls, which may result from the disruption of the BBB caused by the endotoxemia. High-precision isotopic analysis of Mg 24 h after injection of a single high dose of LPS, revealed the disruption of the Mg homeostasis with measurable effects in blood plasma, urine, liver, and brain stem.

## Data Availability Statement

The original data presented in the study are included in the article/Supplementary Materials, further inquiries can be directed to the corresponding author.

## Ethics Statement

The animal study was reviewed and approved by the Ethical Committee for Animal Welfare of the Faculty of Sciences at Ghent University.

## Author Contributions

RG wrote the initial manuscript, performed the measurements, compiled, analyzed, and visualized the data. MC-R designed the concept of the research, supervised the project, and reviewed the manuscript. EV took care of sample collection. RV contributed to the design of the study and reviewed the manuscript. FV ensured funding, supervised the project, and reviewed the manuscript. All authors contributed to the discussion and interpretation of the results and proofread the manuscript.

## Conflict of Interest

The authors declare that the research was conducted in the absence of any commercial or financial relationships that could be construed as a potential conflict of interest.

## Publisher's Note

All claims expressed in this article are solely those of the authors and do not necessarily represent those of their affiliated organizations, or those of the publisher, the editors and the reviewers. Any product that may be evaluated in this article, or claim that may be made by its manufacturer, is not guaranteed or endorsed by the publisher.
