# Supplementary material for: Effect of Endotoxemia Induced by Intraperitoneal Injection of Lipopolysaccharide on the Mg isotopic Composition of Biofluids and Tissues in Mice
Source: Front Med (Lausanne). 2021 Jul 23;8:664666. doi: 10.3389/fmed.2021.664666 (PMC8342922; doi:10.3389/fmed.2021.664666)
Supplement: Supplementary file 1 [file Table_1.DOCX]

Supplementary Material

**Table S1** Concentrations of minor elements (10^2^ µg g^-1^) in organs and urine of aged controls and LPS-injected mice. Number of individuals per group is 3 for controls and 4 for LPS-injected mice; for urine, the number of individuals is 2 for controls and 3 for LPS-injected mice.

| **Sample** | | **Element** | **Control mice** | |  | **LPS-injected mice** | |  | **P value** |
| --- | --- | --- | --- | --- | --- | --- | --- | --- | --- |
|  |  |  | **Mean** | **SD** |  | **Mean** | **SD** |  |  |
| **Bone (femur)** | | Ca | 1200 | 620 |  | 720 | 200 |  | ns. |
|  |  | Mg | 22 | 12 |  | 15 | 4.6 |  | ns. |
|  |  | Na | 38 | 21 |  | 20 | 8.9 |  | ns. |
|  |  | P | 440 | 250 |  | 195 | 79 |  | ns. |
|  |  | S | 1.11 | 0.97 |  | 1.6 | 1.5 |  | ns. |
|  |  |  |  |  |  |  |  |  |  |
| **Intestine** |  | Ca | 0.96 | 0.11 |  | 1.94 | 0.81 |  | ns. |
|  |  | Mg | 1.467 | 0.038 |  | 1.670 | 0.073 |  | 0.007** |
|  |  | Na | 16.9 | 4.3 |  | 17.5 | 1.3 |  | ns. |
|  |  | P | 15.96 | 0.69 |  | 20.5 | 4.5 |  | ns. |
|  |  | S | 1.84 | 0.21 |  | 2.63 | 0.83 |  | ns. |
|  |  |  |  |  |  |  |  |  |  |
| **Kidney** |  | Ca | 0.243 | 0.016 |  | 0.42 | 0.15 |  | ns. |
|  |  | Mg | 1.37 | 0.21 |  | 1.535 | 0.049 |  | ns. |
|  |  | Na | 11.8 | 3.0 |  | 9.58 | 0.66 |  | ns. |
|  |  | P | 16.9 | 2.7 |  | 13.6 | 1.0 |  | ns. |
|  |  | S | 1.89 | 0.31 |  | 1.69 | 0.14 |  | ns. |
|  |  |  |  |  |  |  |  |  |  |
| **Liver** |  | Ca | 0.210 | 0.007 |  | 0.30 | 0.14 |  | ns. |
|  |  | Mg | 1.65 | 0.25 |  | 2.07 | 0.58 |  | ns. |
|  |  | Na | 7.0 | 2.2 |  | 8.2 | - |  | ns. |
|  |  | P | 17.3 | 3.6 |  | 14.8 | 4.1 |  | ns. |
|  |  | S | 2.30 | 0.53 |  | 1.82 | 0.33 |  | ns. |
|  |  |  |  |  |  |  |  |  |  |
| **Muscle** |  | Ca | 0.51 | 0.31 |  | 0.71 | 0.30 |  | ns. |
|  |  | Mg | 1.70 | 0.38 |  | 2.1 | 1.0 |  | ns. |
|  |  | Na | 7.4 | 3.5 |  | 1.79 | 0.87 |  | ns. |
|  |  | P | 11.5 | 2.4 |  | 11.1 | 3.0 |  | ns. |
|  |  | S | 2.17 | 0.61 |  | 2.56 | 0.86 |  | ns. |
|  |  |  |  |  |  |  |  |  |  |
|  |  |  |  |  |  |  |  |  |  |
| **Pancreas** |  | Ca | 0.77 | 0.21 |  | 1.17 | 0.30 |  | ns. |
|  |  | Mg | 2.83 | 0.61 |  | 2.69 | 0.29 |  | ns. |
|  |  | Na | 14.15 | 0.91 |  | 13.70 | 0.99 |  | ns. |
|  |  | P | 34.62 | 5.07 |  | 22.98 | 7.11 |  | ns. |
|  |  | S | 2.09 | 0.41 |  | 2.11 | 0.24 |  | ns. |
|  |  |  |  |  |  |  |  |  |  |
| **Urine** |  | Ca | 0.75 | 0.22 |  | 0.32 | 0.19 |  | ns. |
|  |  | Mg | 9.0 | 1.3 |  | 2.8 | 2.4 |  | 0.050* |
|  |  | Na | 27.1 | 1.7 |  | 0.57 | - |  | - |
|  |  | P | 8.1 | 5.6 |  | 12.8 | 8.0 |  | ns. |
|  |  | S | 2.60 | 0.60 |  | 1.55 | 0.68 |  | ns. |

*Significant difference between controls and LPS-injected mice at p < 0.05 (independent samples t-test)

**Table S2** Concentrations of minor elements (10^2^ µg g^-1^) in organs and urine of young controls and LPS-injected mice. Number of individuals per group is 3.

| **Sample** | | **Element** | **Control mice** | |  |  | **LPS-injected mice** | |  | **P value** |
| --- | --- | --- | --- | --- | --- | --- | --- | --- | --- | --- |
|  |  |  | **Mean** | **SD** |  |  | **Mean** | **SD** |  |  |
| **Bone (femur)** | | Ca | 1249 | 255 |  |  | 1358 | 29 |  | ns. |
|  |  | Mg | 24.46 | 4.60 |  |  | 27.06 | 0.98 |  | ns. |
|  |  | Na | 44.8 | 6.1 |  |  | 42.8 | 2.5 |  | ns. |
|  |  | P | 420 | 75 |  |  | 510 | 60 |  | ns. |
|  |  |  |  |  |  |  |  |  |  |  |
| **Intestine** |  | Ca | 1.60 | 1.32 |  |  | 1.16 | 0.68 |  | ns. |
|  |  | Mg | 1.89 | 0.54 |  |  | 1.77 | 0.18 |  | ns. |
|  |  | Na | 18.5 | 3.4 |  |  | 19.7 | 1.5 |  | ns. |
|  |  | P | 16.4 | 4.8 |  |  | 14.4 | 1.6 |  | ns. |
|  |  | S | 1.09 | 0.74 |  |  | 2.10 | 0.24 |  | ns. |
|  |  |  |  |  |  |  |  |  |  |  |
| **Kidney** |  | Ca | 0.261 | 0.051 |  |  | 0.43 | 0.24 |  | ns. |
|  |  | Mg | 2.03 | 0.11 |  |  | 1.708 | 0.075 |  | 0.014* |
|  |  | Na | 17.1 | 2.5 |  |  | 15.8 | 1.0 |  | ns. |
|  |  | P | 24.7 | 1.0 |  |  | 19.4 | 0.1 |  | 0.001** |
|  |  | S | 2.641 | 0.092 |  |  | 2.25 | 0.10 |  | 0.008** |
|  |  |  |  |  |  |  |  |  |  |  |
| **Liver** |  | Ca | 0.110 | 0.024 |  |  | 0.195 | 0.018 |  | 0.009** |
|  |  | Mg | 2.046 | 0.074 |  |  | 2.05 | 0.10 |  | ns. |
|  |  | Na | 6.9 | 0.7 |  |  | 9.2 | 1.5 |  | ns. |
|  |  | P | 19.9 | 1.1 |  |  | 19.5 | 0.7 |  | ns. |
|  |  | S | 2.50 | 0.13 |  |  | 2.351 | 0.056 |  | ns. |
|  |  |  |  |  |  |  |  |  |  |  |
| **Muscle** |  | Ca | 0.65 | 0.27 |  |  | 0.71 | 0.13 |  | ns. |
|  |  | Mg | 2.16 | 0.08 |  |  | 2.83 | 0.76 |  | ns. |
|  |  | Na | 16.0 | 0.9 |  |  | 11.1 | 6.0 |  | ns. |
|  |  | P | 12.5 | 1.1 |  |  | 16.6 | 5.8 |  | ns. |
|  |  | S | 2.72 | 0.18 |  |  | 3.23 | 0.65 |  | ns. |
|  |  |  |  |  |  |  |  |  |  |  |
|  |  |  |  |  |  |  |  |  |  |  |
| **Pancreas** |  | Ca | 1.23 | 0.54 |  |  | 1.05 | 0.21 |  | ns. |
|  |  | Mg | 2.61 | 0.35 |  |  | 2.75 | 0.23 |  | ns. |
|  |  | Na | 15.0 | 2.1 |  |  | 12.5 | 0.5 |  | ns. |
|  |  | P | 30.1 | 4.1 |  |  | 30.9 | 2.4 |  | ns. |
|  |  | S | 1.72 | 0.50 |  |  | 2.18 | 0.11 |  | ns. |
|  |  |  |  |  |  |  |  |  |  |  |
| **Urine** |  | Ca | 0.61 | 0.67 |  |  | 1.32 | 1.33 |  | ns. |
|  |  | Mg | 7.88 | 1.78 |  |  | 1.48 | 0.50 |  | 0.004** |
|  |  | Na | 23.8 | 10.3 |  |  | 10.2 | 10.7 |  | ns. |
|  |  | P | 4.2 | 5.7 |  |  | 7.7 | - |  | - |
|  |  | S | 1.52 | 0.58 |  |  | 1.39 | 0.56 |  | ns. |

*Significant difference between controls and LPS-injected mice at p < 0.05 (independent samples t-test)

**Significant difference between controls and LPS-injected mice at p < 0.01 (independent samples t-test)

**Table S3** Concentrations of minor elements (µg g^-1^) in different brain regions (cerebellum, hippocampus, brain stem, cortex and cerebrum) of young and aged LPS-injected mice and matched controls. The p-value corresponds to the level of significance between controls and LPS-injected mice.

| **Sample** |  | **Element** | **Controls** | |  | **LPS-injected mice** | |  | **p value** |
| --- | --- | --- | --- | --- | --- | --- | --- | --- | --- |
|  |  |  | **Mean** | **SD** |  | **Mean** | **SD** |  |  |
| ***Young mice*** | |  |  |  |  |  |  |  |  |
| **Cerebellum** | | Ca | 83 | 65 |  | 96 | 97 |  | ns. |
|  |  | K | 2730 | 110 |  | 2730 | 340 |  | ns. |
|  |  | Mg | 144.5 | 6.0 |  | 144 | 13 |  | ns. |
|  |  | Na | 780 | 170 |  | 850 | 380 |  | ns. |
|  |  | P | 2290 | 320 |  | 2360 | 380 |  | ns. |
|  |  | S | 444 | 17 |  | 500 | 190 |  | ns. |
|  |  |  |  |  |  |  |  |  |  |
| **Hippocampus** | | Ca | 340 | 260 |  | 271 | 80 |  | ns. |
|  |  | K | 1960 | 1100 |  | 2580 | - |  | ns. |
|  |  | Mg | 219 | 97 |  | 209 | 52 |  | ns. |
|  |  | Na | 1364 | 56 |  | 730 | 750 |  | ns. |
|  |  | P | 2560 | 960 |  | 2110 | 140 |  | ns. |
|  |  | S | 420 | 120 |  | 467 | 11 |  | ns. |
|  |  |  |  |  |  |  |  |  |  |
| **Brain stem** | | Ca | 218 | 55 |  | 310 | 280 |  | ns. |
|  |  | K | 2620 | 370 |  | 2220 | 980 |  | ns. |
|  |  | Mg | 159 | 32 |  | 152 | 26 |  | ns. |
|  |  | Na | 1140 | 720 |  | 660 | 480 |  | ns. |
|  |  | P | 2540 | 610 |  | 2730 | 490 |  | ns. |
|  |  | S | 460 | 130 |  | 450 | 180 |  | ns. |
|  |  |  |  |  |  |  |  |  |  |
| **Cortex** | | Ca | 193 | 63 |  | 62.3 | - |  | ns. |
|  |  | K | 1580 | 800 |  | 2230 | 880 |  | ns. |
|  |  | Mg | 155.3 | 1.3 |  | 173 | 42 |  | ns. |
|  |  | Na | 1350 | 170 |  | 880 | 490 |  | ns. |
|  |  | P | 1840 | 460 |  | 2180 | 690 |  | ns. |
|  |  | S | 445 | 75 |  | 300 | 260 |  | ns. |
|  | |  |  |  |  |  |  |  |  |
|  | |  |  |  |  |  |  |  |  |
| **Cerebrum** | | Ca | 134 | 63 |  | 54.7 | 5.6 |  | ns. |
|  |  | Mg | 168.4 | 2.5 |  | 179 | 15 |  | ns. |
|  |  | Na | 1113 | 54 |  | 1197 | 91 |  | ns. |
|  |  | P | 2415 | 70 |  | 2523 | 81 |  | ns. |
|  |  | S | 195 | 1 |  | 208 | 6 |  | 0.021* |
|  |  |  |  |  |  |  |  |  |  |
| ***Aged mice*** | |  |  |  |  |  |  |  |  |
| **Cerebellum** | | Ca | 76 | 50 |  | 88 | 59 |  | ns. |
|  |  | K | 2520 | 480 |  | 2960 | 690 |  | ns. |
|  |  | Mg | 155 | 14 |  | 142.4 | 7.4 |  | ns. |
|  |  | Na | 600 | 150 |  | 760 | 370 |  | ns. |
|  |  | P | 2040 | 820 |  | 1920 | 790 |  | ns. |
|  |  | S | 330 | 300 |  | 660 | 250 |  | ns. |
|  |  |  |  |  |  |  |  |  |  |
| **Hippocampus** | | Ca | 119.7 | 57 |  | 56.3 | - |  | ns. |
|  |  | K | 2700 | 170 |  | 3450 | 810 |  | ns. |
|  |  | Mg | 176 | 31 |  | 139 | 17 |  | ns. |
|  |  | Na | 1120 | 1300 |  | 1200 | 1300 |  | ns. |
|  |  | P | 1940 | 540 |  | 1670 | 96 |  | ns. |
|  |  | S | 443 | 96 |  | 1000 | 810 |  | ns. |
|  |  |  |  |  |  |  |  |  |  |
| **Brain stem** | | Ca | 33 | 13 |  | 77.9 | 7.0 |  | 0.007** |
|  |  | K | 1930 | 300 |  | 2950 | 430 |  | 0.014* |
|  |  | Mg | 147 | 13 |  | 149.7 | 6.2 |  | ns. |
|  |  | Na | 900 | 410 |  | 1360 | 470 |  | ns. |
|  |  | P | 2620 | 120 |  | 2540 | 340 |  | ns. |
|  |  | S | 430 | 120 |  | 920 | 320 |  | 0.033* |
|  |  |  |  |  |  |  |  |  |  |
| **Cortex** | | Ca | 42 | 19 |  | 43.5 | - |  | ns. |
|  |  | K | 1900 | 900 |  | 3320 | 200 |  | ns. |
|  |  | Mg | 136 | 12 |  | 144 | 10 |  | ns. |
|  |  | Na | 650 | 110 |  | 1860 | 1000 |  | ns. |
|  |  | P | 1560 | 380 |  | 2240 | 310 |  | ns. |
|  |  | S | 338 | 35 |  | 1230 | 680 |  | ns. |
|  |  |  |  |  |  |  |  |  |  |
| **Cerebrum** | | Ca | 46 | 32 |  | 53 | 18 |  | ns. |
|  |  | Mg | 147.0 | 1.1 |  | 154.8 | 8.4 |  | ns. |
|  |  | Na | 830 | 150 |  | 751.4 | 2.7 |  | ns. |
|  |  | P | 1890 | 250 |  | 1580 | 190 |  | ns. |

ns. = difference is not significant
