# Supplementary figures and images for: Effect of Endotoxemia Induced by Intraperitoneal Injection of Lipopolysaccharide on the Mg isotopic Composition of Biofluids and Tissues in Mice
Source: Front Med (Lausanne). 2021 Jul 23;8:664666. doi: 10.3389/fmed.2021.664666 (PMC8342922; doi:10.3389/fmed.2021.664666)

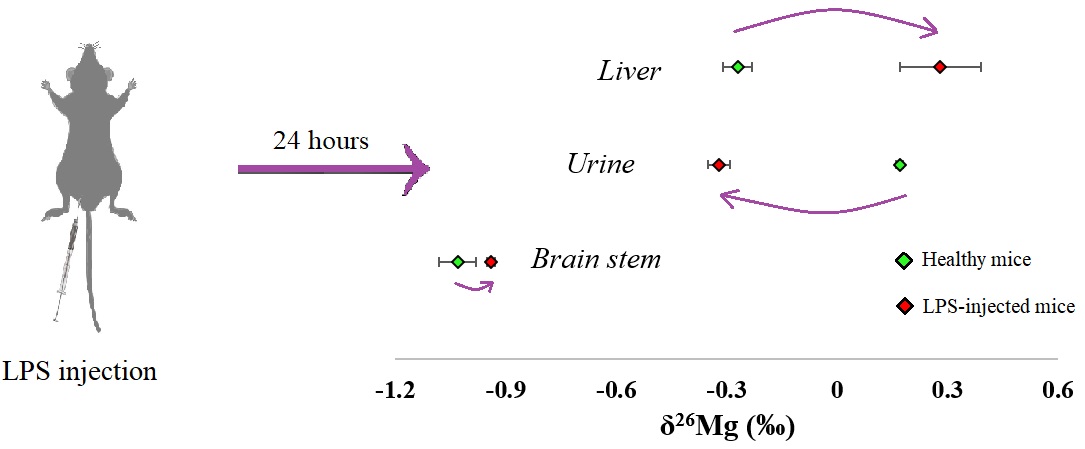

Supplement: Supplementary file 2 [file Image_1.JPEG]
